# Michigan State Medical Association

**Published:** 1849-05

**Authors:** 


*Michigan State Medical Association.*—The following report of the proceedings of a Convention lately held in Jackson, Michigan, which resolved itself into an Association, has been transmitted to us for publication. In the letter which accompanied it, the writer states, that "a general feeling of interest is awakened throughout the State on Medical subjects. I think our Convention will do something towards elevating the profession. Its formation has been hailed with pleasure by Medical gentlemen over the State." We heartily wish success to the efforts of our brethren who have been active in this movement.

JACKSON, Jan. 3d, 1839.

At a meeting of delegates to attend the State Medical Convention, called by the Genessee Co., and Jaekson Co., Medical Societies, for the improvement and elevation of the Medical Profession in this State—Dr. John Cadman, of Lenawee, was appointed Chairman, pro tem., and D. Laskie Miller, of Genessee, was chosen Secretary pro tem.

On motion of Dr. Graham, of Genessee, a committee of three was appointed to report a plan for the organization of the Convention.

The Chair appointed Drs. Graham, Eldredge, and Cornell said committee.

On motion of Dr. McNaughton, of Jackson, a committee of three was appointed to receive credentials of delegates, and report the names of gentlemen entitled to seats in the convention, Drs. McNaughton, Backus and Hoyt, were appointed said committee.

On motion of Dr. Eldredge, of Lapeer, the committee on credentials was instructed to report the name of one individual for President, two for Vice Presidents, and two for Secretaries.

On motion of Dr. Fish, of Jackson, the convention adjourned until half past one o'clock, P. M.

—

AFTERNOON SESSION.

The convention met agreeably to adjournment and was called to order by the Chair.

The committee appointed to report a plan for the permanent organization of the convention, through the Chairman, reported a plan for the organization of the *Michigan Medical Association*, which was accepted and adopted.

The committee on credentials read their report, which was accepted.

The convention then proceeded to the election of officers for the ensuing year, which resulted as follows:

For President,—Dr. John Cadman, of Lenawee.

1st Vice President,—N. Buel Eldridge, of Lapeer.

2d Vice President, B. Hard Van Buren.

Secretaries, D. Laskie Miller, Genessee; M. A. McNaughton, Jackson.

Treasurer, D. Sager, Washtenaw.

Dr. Fish offered the following:

Resolved, That it is the duty of Physicians, in their individual capacity, as well as in their associations as County or State Societies, to withhold their patronage, as far as practicable, from those drug stores that are engaged in vending secret nostrums.

Which was discussed freely by Drs. Backus, Graham, Eldredge, and McNaughton, and adopted unanimously.

The President announced the standing committees, as follows:

On arrangements, Drs. Higby, Graham and Snyder.

On practical Medicine, Drs. Sager, Camburn, and Spencer.

On Surgery, Drs. Gunn, Marsh and Douglass.

On Obstertries, Drs. Cornell, J. N. Graham, and Fish.

On Medical Education, Drs. Stetson, Palmer, and Fairbank.

On Publication, Drs. Miller, McNaughton and Sager.

The Association then adopted the code of Medical Ethics adopted by the American Medical Association.

On motion the Association then proceeded to elect five delegates to attend the next meeting of the American Medical Association, which resulted in the election of Drs. N. Buell Eldridge, of Lapeer, M. Gunn, of Washtenaw, John Cadman, of Lenawee, A. Sager, of Washtenaw, and A. B. Palmer, of Lenawee.

On motion of Dr. Palmer.

The delegates to the National Association were authorized to appoint substitutes in case of their inability to attend.

Dr. Eldridge offered the following:

Resolved, That the Physicians of Jackson are entitled to, and do hereby receive our thanks for the kindness extended to those from abroad, while attending the meeting of this Association.

Carried unanimously.

On motion of Dr. Fairbank.

Resolved, That when we adjourn, we adjourn to meet at Ann Arbor on the 3rd Wednesday of January, 1850.

Dr. Graham offered the following:

Resolved, That the President of this Association be requested to deliver an Address at the next meeting of the Association, with power to appoint an alternate—which was adopted.

On motion of Dr. Fish.

An abstract of the proceedings of this meeting, was ordered to be published in the papers of this place.

JOHN CADMAN, *President.*

Appended to the report were the names of the Secretaries of the convention, which have, accidentally, been partially torn off in the copy sent to us, and we are therefore obliged to omit them.

